# Discrete Element Modeling and Electron Microscopy Investigation of Fatigue-Induced Microstructural Changes in Ultra-High-Performance Concrete

**DOI:** 10.3390/ma14216337

**Published:** 2021-10-23

**Authors:** Sebastian Rybczynski, Gunnar Schaan, Maksym Dosta, Martin Ritter, Frank Schmidt-Döhl

**Affiliations:** 1 Institute of Materials, Physics and Chemistry of Buildings, Hamburg University of Technology, 21073 Hamburg, Germany; gunnar.schaan@tuhh.de (G.S.); schmidt-doehl@tuhh.de (F.S.-D.); 2 Institute of Solids Process Engineering and Particle Technology, Hamburg University of Technology, 21073 Hamburg, Germany; dosta@tuhh.de; 3 Electron Microscopy Unit, Hamburg University of Technology, 21073 Hamburg, Germany; ritter@tuhh.de

**Keywords:** ultra-high-performance concrete, fatigue, electron microscopy, ettringite transformation, bonded particle model, discrete element method, rheological model, crack propagation

## Abstract

In view of the growing demand for sustainable and lightweight concrete structures, the use of ultra-high-performance concrete (UHPC) is becoming increasingly important. However, fatigue loads occur more frequently in nature than static loads. Despite the impressive mechanical properties of UHPC, a reduced tolerance for cyclic loading is known. For this reason, our paper deals with experimental and numerical investigations regarding the main causes for crack initiation on the meso, micro, and nanoscale. After mechanical fatigue tests, we use both scanning (SEM) and transmission electron microscopy (TEM) to characterize microstructural changes. A new rheological model was developed to apply those changes to the mesoscopic scale. The origins of fatigue damaging can be traced back to a transformation of nanoscale ettringite, resulting in a densification of the surrounding binder matrix. Additionally, a higher content of unhydrated cement clinker in the matrix benefits fatigue resistance. On the mesoscale, stress peaks around aggregate grains expand into the surrounding binder with increasing load cycles and lead to higher degradation.

## 1. Introduction

Fatigue-induced damage of cementitious structures, similar to concrete foundations of wind turbines or concrete bridges, is more common than static loading in nature. When these structures are subjected to cyclic loading, plastic deformation is observed even though the maximum load level is much smaller than the short-term strength under quasi-static loading conditions. Due to the more frequent use of innovative materials such as ultra-high-performance concrete (UHPC), the focus of research is shifting toward the main crack-initiating factors. UHPC is an almost ideally brittle material with a very dense and high-strength structure. The outstanding mechanical properties are achieved by an optimization on the basis of the maximum packing density theory [1,2] and a very low water to cement ratio (w/c). A high amount of cement, fillers (quartz or limestone powder), and pozzolanic additives such as silica fume lead to the optimized particle size distribution. Due to the low w/c ratio, the degree of cement hydration in UHPC is as low as 30%, so much of the unhydrated cement also acts as physical filler. Furthermore, the pozzolanic reactivity of silica—the additional formation of calcium silicate hydrate (C-S-H)—and its small particle size support a high-strength interfacial transition zone (ITZ) and lead to a lower porosity within the binder [3,4]. As a result, crack propagation from pores or a weaker ITZ cannot be assumed, as it is the case for plain concrete. Recently, investigations regarding crack initiation have concentrated on localizing inhomogeneities, particularly in the case of fatigue loading.

Both scanning (SEM) and transmission electron microscopy (TEM) have been applied to cementitious materials for four decades [5,6,7,8,9], but so far, few if any reports focused on the effects of fatigue on the microstructure of the binder matrix. We aim to investigate these effects, down to their origins on the nanoscale, using SEM as well as scanning TEM on lamella samples prepared using focused ion beam (FIB) techniques. In past research, energy-dispersive X-ray spectroscopy (EDS) applied to cementitious materials has mostly served to determine the local chemical composition of the binder matrix [10,11,12]. However, we additionally employ EDS to detect and identify objects on the nanoscale.

For our numerical investigations, the bonded particle model (BPM) was used. The BPM is an extension of the soft-sphere discrete element method (DEM), where particles can be connected by solid bonds [13,14]. This enables the modeling of porous [15] or coherent materials structures consisting of single or various components. The overall behavior of the material is described by constitutive models for particle interactions and by solid bond models. The possibility to change or remove individual bonds during the simulation allows for the reproduction of small-scale changes or fracture of the material. BPM has been increasingly used by research groups for the numerical analysis of concrete in 2D and 3D simulations during static loading [16,17,18,19,20,21,22], but none of them have applied the BPM to UHPC. Furthermore, only a few models have been developed for cyclic loading [23,24,25].

The main purpose of this article is the illustration of a beneficial application of various experimental and numerical methods to calibrate and concretize a newly derived model approach for fatigue simulations of UHPC. We aim to identify vulnerable elements of the concrete microstructure and to contribute to the development of cementitious building materials with improved fatigue resistance.

A general characterization of components and materials is given in Section 2. Results of SEM and TEM investigations of fatigue-damaged samples are presented in Section 3 and Section 4, respectively. Section 5 introduces the numerical background and proposes a new rheological fatigue model, while Section 6 presents results of fatigue simulations. A short summary and main conclusions are given in Section 7.

## 2. Materials Characterization

Specimens of UHPC and pure binder were prepared from components given in Table 1. Oxide compositions for cement and silica fume are given in the Supplementary Data (Table S1). Specimens were stripped after 24 h and then stored in water until a minimum age of 56 d, since the compressive strength of UHPC still increases considerably after a standard hydration time of 28 d [26]. This procedure ensures a high degree of hydration and a nearly constant compressive strength. Afterwards, the samples were sawn and ground to cylindrical dimensions of h/d = 180 mm/60 mm.

To investigate the mechanical behavior of UHPC and binder specimens, uniaxial static and cyclic compression tests were carried out. Binder samples have the same composition as the UHPC, excluding the quartz sand.

Uniaxial static compression tests were carried out strain-controlled with a load speed of 0.2 mm/min on three specimens of both materials. Three high-precision inductive displacement transducers were used to capture the longitudinal strain during testing with a sampling rate of 30 Hz. The mean values of the mechanical properties are shown in Table 2. Both the strength and stiffness of the binder samples are lower than for UHPC samples, while the breakage strain is higher. This is related to the missing aggregate grains in the mixture. Nevertheless, the main contributing factor to the high static strength of UHPC and binder is the optimized packing density and low w/c ratio, while the aggregate properties play a secondary role.

**Figure 1:**
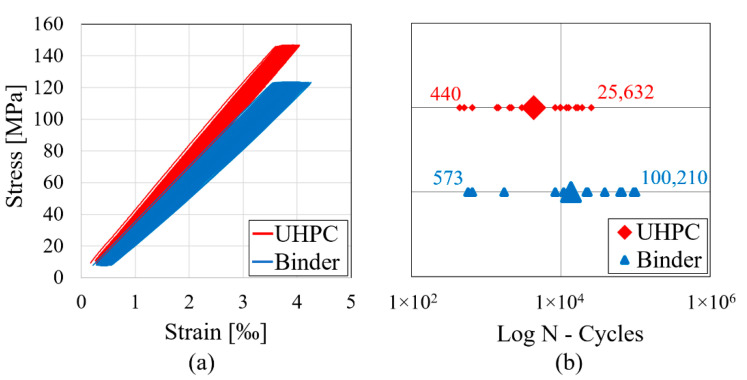
Results of experimental fatigue tests on UHPC and binder: (**a**) Typical stress–strain curves during fatigue; (**b**) Lifetime until fatigue failure (mean and single values).

After static tests, uniaxial cyclic compression tests were carried out force-controlled with a triangular load regime of 1 Hz. The upper and lower load level ($S_{u}$, $S_{l}$) corresponded to 80% and 5% of the mean value of the static strength, while the longitudinal strain was measured by three high-precision inductive displacement transducers with a sampling rate of 30 Hz. Figure 1a shows typical stress–strain curves during fatigue testing for both compositions under the mentioned regime, while Figure 1b contains the lifetime values (single values, mean values, and max/min).

The induced fatigue damage leads to an increase of strain and decrease of stiffness for both compositions (Figure 1a). As it is often the case with brittle materials, no failure announcement could be observed, while an abrupt breakage occurred without any post-breakage behavior. Generally, fatigue tests are always subjected to a high scattering caused by material heterogeneity. Nevertheless, a higher fatigue resistance could be observed for binder specimens (Figure 1b).

## 3. Correlation of Unhydrated Cement Clinker Content and Specimen Lifetime

### 3.1. Sample Preparation

To find a possible correlation between the lifetime of a specimen and its content of unhydrated cement clinker, we performed SEM investigations and image analysis on sufficiently large sample surfaces to ensure statistical reliability. A similar approach to analyze the hydration degree from segmented images to support numerical models has mostly been applied to μCT data [27], but no reports on the application to SEM data are known so far.

Specimen pieces with a surface of approximately 1 to 2 cm² were cut from suitable fragments of after-failure samples using a low-speed diamond blade saw. The surface was finely ground using diamond-coated sandpaper of four different grits down to 8.4 µm and water cooling. Then, these samples were glued to standard aluminum SEM carriers and coated with a thin electrically conductive layer using carbon evaporation.

### 3.2. SEM Investigation and Image Processing

Material or atomic number (*Z*) contrast imaging of backscattered electrons (BSE) with a concentric ring detector was employed to easily discern between aggregate, binder matrix, and unhydrated cement clinker. To survey and analyze large surface areas, individual images with a horizontal field width of ca. 1 mm and a pixel size of below 1 µm were acquired in a serpentine pattern, overlapping by 20% in both the x and y directions. The individual images were fused using a FIJI/ImageJ plugin [28] to create a single large-area image of each sample. These were cropped to exclude specimen edges, covering 8000×8000 pixels (ca. 5.6 × 5.6 mm²) in total.

To improve the image quality and reliability of analysis, several image processing steps were performed:

1. Non-local means denoising (σ = 20) [29,30] to reduce noise, especially in homogeneous parts of the specimen surface, such as aggregate grains;
2. Band-pass filter with lower and upper limits of 3 and 5000 pixels, respectively, to suppress brightness gradients caused by an imperfectly plan-parallel specimen surface;
3. K-means clustering [31,32,33] to segment the image into four individual clusters, corresponding to, from the darkest to brightest: cracks and pore edges, aggregate grains, hydrated binder matrix, and unhydrated cement clinker grains;
4. Gray-level thresholding to select desired components and particle analysis (10 pixels or larger) to exclude negligibly small particles stemming from residual image noise.

**Figure 2:**
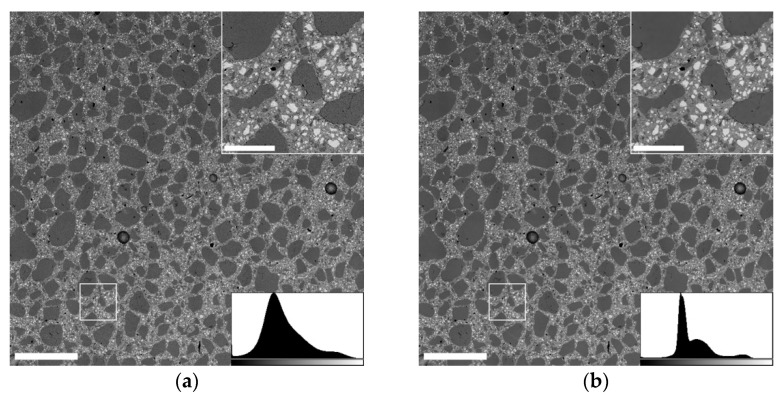

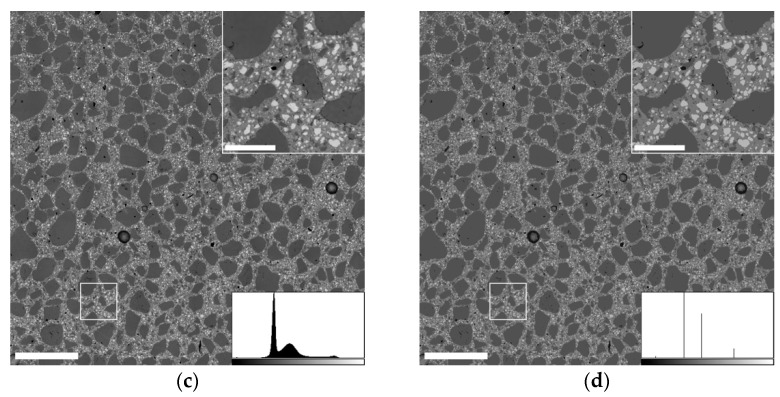
Steps of SEM image processing and segmentation: (**a**) Unprocessed image; (**b**) After non-local means denoising; (**c**) After band-pass filtering; (**d**) After k-means clustering segmentation. Scale bars in large-area images and higher-magnification insets are 1 mm and 200 µm, respectively.

Exemplary large-area SEM images, corresponding gray-level histograms, and higher-magnification insets of the marked area are shown in Figure 2.

### 3.3. Area Calculation and Analysis

The respective area fractions of the individual components in the images were considered a sufficiently accurate approximation of their content in the specimen volume. The relative content *x* of unhydrated cement clinker in the binder matrix was calculated as a ratio of unhydrated cement clinker area $A_{\mathrm{clinker}}$ (taken directly from particle analysis) and binder area $A_{\mathrm{binder}}$ (total image area A without aggregate area $A_{\mathrm{aggregate}}$ from particle analysis):

(1) $$x=\frac{A_{\mathrm{clinker}}}{A_{\mathrm{binder}}}=\frac{A_{\mathrm{clinker}}}{A-A_{\mathrm{aggregate}}}$$

**Figure 3:**
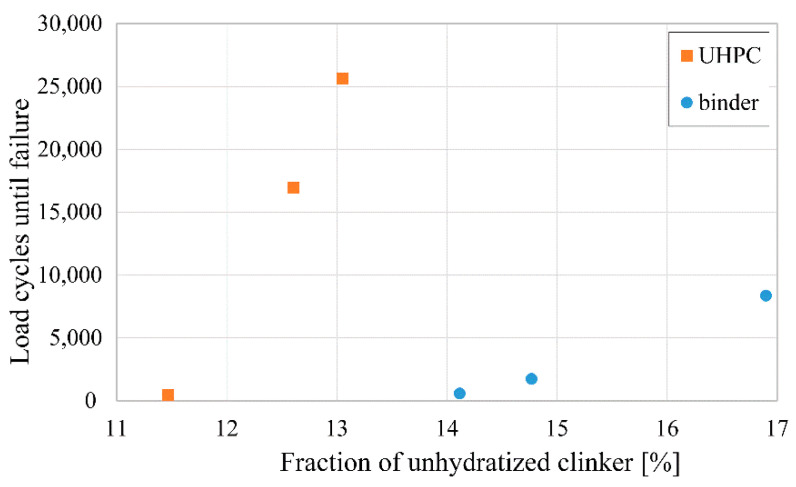
Graph of specimen lifetime vs. their relative content of unhydrated cement clinker showing a positive correlation.

We find a positive correlation between the relative content of unhydrated cement clinker in a specimen and its eventual lifetime, the effect being more pronounced for UHPC samples due to their lower total binder content. The corresponding graph is shown in Figure 3. A high content of unhydrated cement clinker, which can be considered a high-strength ceramic material, lowers local w/c ratios and in turn increases local strength.

## 4. Characterization of Structural Changes on the Nanoscale

The lasting strain introduced with every single load cycle must be connected to irreversible changes in the nanoscale structure of the material. In a recent publication [34], we used TEM for this purpose and observed needle-shaped objects forming in the binder matrix of UHPC already after the early onset of fatigue damage. These objects retain their projected size of ca. 200 nm in length and ca. 30 nm in width but continuously increase in number as fatigue damage progresses.

Due to their increasingly dark contrast with respect to the surrounding material in *Z*-contrast high-angle annular dark field (HAADF) STEM imaging, we initially concluded these needle-shaped structural changes to be crack precursors, continuously depleting in material while the surrounding matrix is densified.

**Figure 4:**
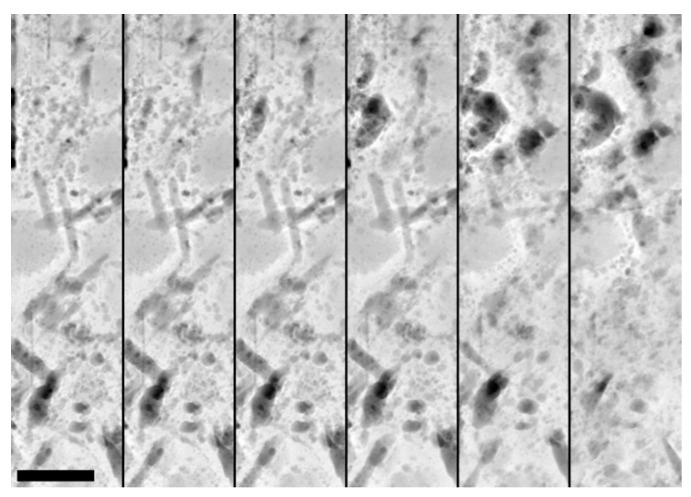
Sections of HAADF-STEM images after increasing durations of electron irradiation showing both shrinking and growing structural changes. From left to right: after 6 s; 30 s; 1 min; 2 min; 5 min; 10 min. Scale bar is 200 nm.

**Figure 5:**
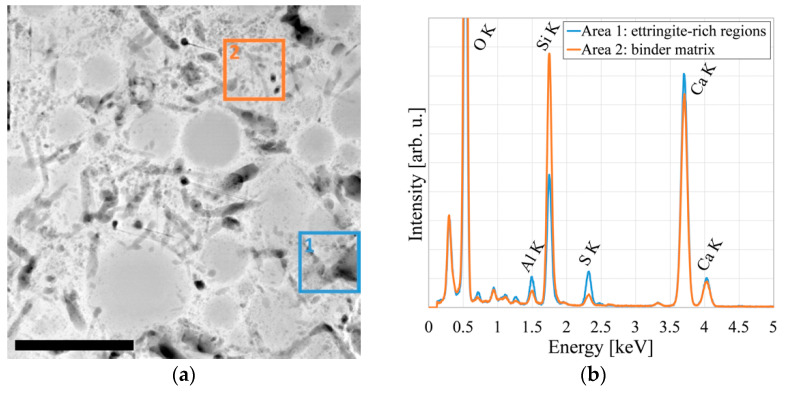

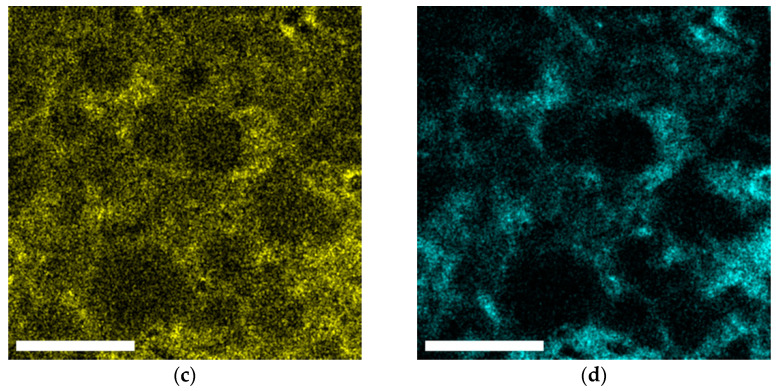
Ettringite transformation in the binder matrix after early-stage fatigue: (**a**) HAADF-STEM image indicating where spectra were extracted; (**b**) Corresponding EDS spectra; (**c**) EDS map of aluminum distribution; (**d**) EDS map of sulfur distribution. Scale bar is 500 nm.

Upon further investigation, we find that the structural changes (1) either shrink or grow in size upon energy uptake from electron irradiation during TEM investigation and (2) preferentially exist in regions of the binder matrix comparatively rich in aluminum and sulfur and with a low silicon content. A selection of HAADF-STEM image sections after increasing irradiation durations is shown in Figure 4; EDS elemental distribution maps and spectra are displayed in Figure 5.

As these structural changes exhibit dark *Z* contrast in HAADF-STEM imaging, readily change size and shape upon energy uptake, and preferentially exist in regions rich in aluminum and sulfur, we conclude they are connected to locations of a transformation of ettringite.

Ettringite is a mineral with the nominal chemical formula Ca_6_Al_2_(SO_4_)_3_(OH)_12_ · 26 H_2_O. It is rarely found in nature but plays a crucial role in the early stage of the setting of concrete, forming as a result of the reaction of calcium aluminate and calcium sulfate from Portland cement. A delayed formation of ettringite in hardened concrete can be detrimental to the durability and longevity of the material [35]. Due to its high water content of 26 molecules per formula unit, its average atomic number is considerably lower than for other components of UHPC, resulting in a darker image contrast in HAADF-STEM imaging.

For the same reason, its density is low for a crystalline solid (1.77 g/cm³) and its molar volume is up to four times higher than that of C-S-H phases that constitute the majority of the binder matrix. Ettringite is known to readily recrystallize upon changes in environmental parameters, forming coarse, rigid crystals. It is commonly detectable on fracture surfaces and cracks in damaged concrete samples [36]. A recurring dissolution and crystallization can exert pressure on the surrounding material, leading to solidification and, eventually, the formation of cracks on the nanoscale.

**Figure 6:**
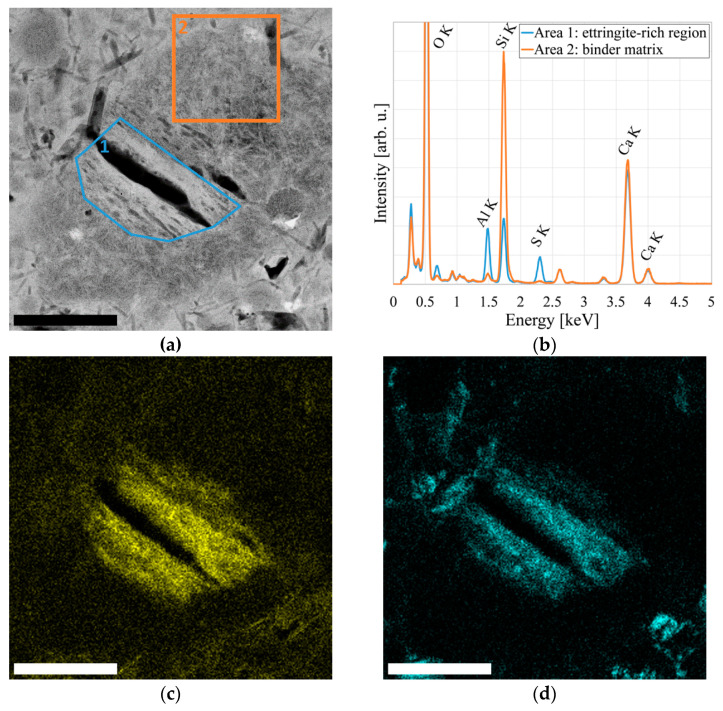
Sample region showing strong ettringite transformation around a nanoscale crack: (**a**) HAADF-STEM image indicating where spectra were extracted; (**b**) Corresponding EDS spectra; (**c**) EDS map of aluminum distribution; (**d**) EDS map of sulfur distribution. Scale bar is 500 nm.

Sample regions containing nanoscale cracks found in after-failure samples exhibit especially high contents of aluminum and sulfur and a very low silicon content, underlining the connection between fatigue damage and nanoscale ettringite transformation. HAADF-STEM images as well as EDS spectra and elemental distribution maps of one such region are displayed in Figure 6.

## 5. Numerical Background

### 5.1. Discrete Element Method

The discrete element method is mainly used to discretize granular materials as an assemblage of primary particles, having their own physical properties and motions. Particles interact with each other and with defined boundary walls, with forces between those interactions being governed by contact models. The motion of particles is described by Newton’s second law, and the acceleration is time-integrated to find the new velocities and positions. Initially, DEM was developed for the modeling of systems consisting of spherical particles [37], but nowadays, several extensions of DEM are used for non-spherical particles, the modeling of multiphase flows [38], or chemical reactions. The simulations presented in this contribution have been performed in the open-source DEM simulation framework MUSEN [39]. The latest version of the code can be downloaded from [40]. The calculation of forces and integration of motion is performed on a graphic processor unit (GPU), which reduces the computation time significantly. The current study has been performed on a computer equipped with Intel Xeon Gold 5,118 CPU and NVIDIA A100 GPU.

### 5.2. Bonded-Particle Model

The bonded particle model is an extension of the DEM, where each pair of the mentioned primary particles can be connected by a solid bond [13,14]. These bonds make it possible to describe a coherent materials structure consisting of single or various components. The overall behavior of the material is described by the interparticle contacts and additional bond models. The possibility to remove individual solid bonds during the simulation allows explicitly simulating the inner fracturing of the material. In this work, a soft-sphere DEM formulation has been used where occurring interparticle or particle–wall overlaps can be seen as local material deformations. Overall, three different types of interactions have been applied in the BPM model:

- Interparticle contacts: calculation of forces between all primary particles even if they are connected with solid bonds;
- Particle–wall contacts: interaction between compression walls and primary particles;
- Solid bonds: forces, moments, and torques acting in single bonds between two particles.

### 5.3. Contact Models

Due to the almost ideally elastic behavior of UHPC and binder, models with an elastic base were chosen for all three mentioned types of interactions. For the interparticle model, a linear elastic formulation was used, while the Hertz–Mindlin model was chosen for the particle–wall contact. A detailed description of these models can be found in [41,42,43]. With respect to the rheological bond model, a newly derived approach was implemented, which uses elastic and plastic deformations as well as fatigue damaging. Basic equations for the purely elastic sections, which also find application in our model, can be found in [13]. However, the main focus of the following description is on the plastic and fatigue damaging of a bond.

**Figure 7:**
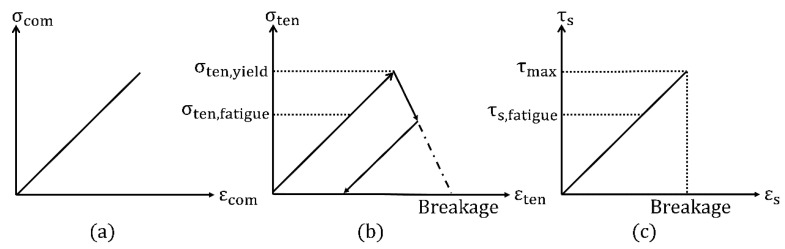
Bond response in different load modes: (**a**) Compression; (**b**) Tension; (**c**) Shear.

Since concrete is known to fail due to internal transverse tensile stresses, the main damaging mechanisms were integrated into the tensile response of the model. Additionally, to capture fatigue degradation from shear stresses, a fatigue damage was also integrated into shear mode. Note that bonds do not break or undergo any damage during pure compression. Figure 7 shows the rheological models for all three load types.

Generally, the stress acting on a bond is a result of the interaction between the corresponding particles. Solid bonds are modeled as ideally cylindrical objects with an initial diameter $d_{\mathrm{init}}$, which cannot exceed the minimal diameter of the connected particles. Depending on the particle movement or rather the relative velocity ${\vec{v}}_{\mathrm{rel}}$, different stresses are acting. In normal direction (compression and tension), the increment of strain $\Delta \varepsilon_{\mathrm{com},\mathrm{ten}}$ and stress $\Delta \sigma_{\mathrm{com},\mathrm{ten}}$ is calculated by Equations (2) and (3), while the sum of the increments yields to the current stress value $\sigma_{\mathrm{current}}$:

(2) $$\;\Delta \varepsilon_{\mathrm{com},\mathrm{ten}}=\frac{v_{\mathrm{rel},n}\cdot \Delta t}{L_{\mathrm{init}}}$$

(3) $$\Delta \sigma_{\mathrm{com},\mathrm{ten}}=\;\Delta \varepsilon \;\cdot \;E_{b}\;$$

where $v_{\mathrm{rel},n}$ is the relative velocity of the particles in normal direction, Δt is the time step, $E_{b}$ is the bond Young’s modulus, and $L_{\mathrm{init}}$ is the initial bond length. The used indices for the stress and strain increments represent the respective load type (compression, tension, shear) and the load direction (normal or shear). Once the tensile yield limit $\sigma_{\mathrm{ten},\mathrm{yield}}$ is exceeded, softening occurs, and the bond starts to deform plastically. Then, the increment of the stress $\Delta \sigma_{\mathrm{ten}}$ is calculated using Equation (4), where α is the weakening factor between the elastic and plastic section of the tensile bond response:

(4) $$\;\Delta \sigma_{\mathrm{ten}}=-\;\Delta \varepsilon_{\mathrm{ten}}\cdot E_{b}\cdot \alpha .$$

**Figure 8:**
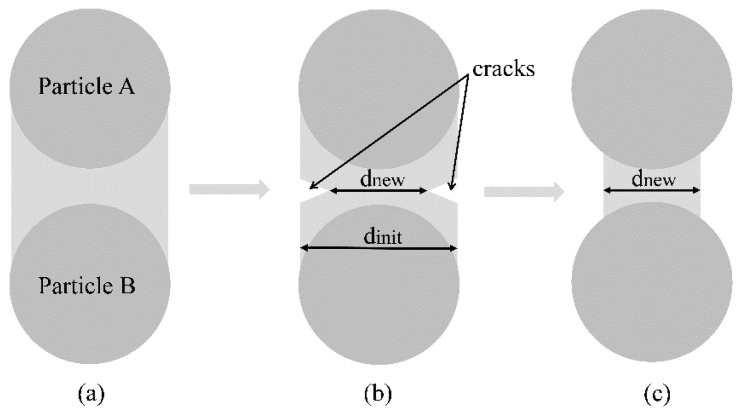
Bond damage caused by a small-scale crack and its implementation: (**a**) Undamaged bond connection of two primary particles; (**b**) A fatigue crack manifests in a reduction of the load-bearing cross-cut of a meso bond; (**c**) Representation of a fatigue crack in the model by a reduced bond diameter.

In the case of fatigue loading of UHPC, crack propagation occurs on different scales and does not inevitably lead to plastic deformation on the mesoscale. For this reason, it is not sufficient to reproduce fatigue degradation only by a plastic model formulation. Instead, crack growth takes place on all small scales (meso, micro, and nanoscale) simultaneously, which has to be taken into account. To capture this on the mesoscale, a fatigue damage criterion was implemented, where the bond diameter is reduced as soon as fatigue damage occurs. Using this approach, the mentioned micro and nanocracks can be represented without having a bond to break directly and be removed from the simulation. Figure 8 illustrates the connection between small-scale damage and its implementation on the mesoscale bond.

Fatigue damage occurs in the elastic range and thus is separated from plastic deformation, which is important in the case of fatigue mechanisms. Therefore, the bond diameter is determined by the stresses acting on two consecutive simulation time steps. The current and previous stress values $\sigma_{\mathrm{ten},\mathrm{cur}}\;$and $\sigma_{\mathrm{ten},\mathrm{prev}}$ are analyzed and compared to the critical value $\sigma_{\mathrm{ten},\mathrm{fatigue}}$, which defines the fatigue level in the elastic section:

(5) $$\sigma_{\mathrm{ten},\mathrm{prev}}>\sigma_{\mathrm{ten},\mathrm{fatigue}}\;\;;\;\;\sigma_{\mathrm{ten},\mathrm{prev}}<\sigma_{\mathrm{ten},\mathrm{yield}}\;$$

(6) $$\sigma_{\mathrm{ten},\mathrm{cur}}<\sigma_{\mathrm{ten},\mathrm{fatigue}}.$$

If the conditions (5) and (6) are fulfilled, then the bond diameter is reduced to a new diameter according to Equation (7), and very small destructions in the supporting material structure are taken into account, as also shown in Figure 8. The larger the value for the damaging factor $\delta_{n}\;$, the smaller the represented micro or nanocrack in the bond:

(7) $$d_{\mathrm{new}}=d_{\mathrm{init}}\cdot \;\left(1-10^{-\delta_{n}}\right).$$

To capture fatigue damage under tension and shear load, a similar criterion was additionally implemented in the shear response of the model. Therefore, the bond diameter is linked with the current and previous stress value $\tau_{s,\mathrm{cur}}\;$and $\tau_{s,\mathrm{prev}}$, while $\tau_{s,\mathrm{fatigue}}$ defines the critical fatigue level:

(8) $$\tau_{s,\mathrm{prev}}>\tau_{s,\mathrm{fatigue}}\;\;;\;\;\tau_{s,\mathrm{prev}}<\tau_{\max}$$

(9) $$\tau_{s,\mathrm{current}}<\tau_{s,\mathrm{fatigue}}.$$

If conditions (8) and (9) are fulfilled, then the bond diameter is corrected to a new diameter according to Equation (10). The larger the value for the damaging factor $\delta_{s}$, the smaller the represented micro or nanocrack in the bond:

(10) $$d_{\mathrm{new}}=d_{\mathrm{init}}\cdot \;\left(1-10^{-\delta_{S}}\right).$$

In order to simulate material destruction, total stresses including additional bending and torsional moments are analyzed and compared with specified material properties for normal-tension strength $\sigma_{\mathrm{ten},\max}$ as well as shear strength $\tau_{\max}$. If one of the conditions in Equations (11) or (12) is fulfilled, the bond breaks and is removed from the simulation:

(11) $$\tau_{s,\mathrm{current}}+M_{t,b\;}\cdot \frac{d_{\mathrm{new}}}{2\cdot I_{T,b}}>\tau_{\max}$$

(12) $$\;\;\;\sigma_{\mathrm{current}}+M_{n,b\;}\cdot \frac{d_{\mathrm{new}}}{2\cdot J_{b}}>\sigma_{t,\;\max}$$

where $M_{t,b\;}$and $M_{n,b\;}$are the torsional and bending moments acting on the bond, $J_{b}\;$is the moment of inertia, and $I_{T,b}\;$is the torsional moment of inertia.

### 5.4. Structural UHPC Model and Calibration of Parameters

With respect to the numerical investigation of the fatigue behavior of UHPC, a structural three-phase UHPC model was used. The model consists of two types of particles (binder and aggregate) and three types of bonds (binder–binder, binder–aggregate, and aggregate–aggregate). These three types of bonds represent the main three phases of concrete (binder, interfacial transition zone, and aggregate) and allow to distinguish and analyze different sections of the modeled UHPC. To consider a realistic non-spherical shape of aggregate, reconstructed 3D models from X-ray computer tomography (µCT) were used for the structural model. The bond formulations presented in Section 5.2 were implemented for the binder and ITZ bonds, while a purely elastic model was used for the aggregate bonds. A detailed procedure of the used algorithms on which the structure generation is based can be found in [41], and only the most significant points will be mentioned here as follows.

Caused by the optimized packing density of the used UHPC composition, a potentially weaker ITZ was not observed in SEM investigations [41]. For this reason, parameters of ITZ and binder bonds were assumed to be equal. This reduced the calibration effort to the remaining phases of binder and aggregate. With respect to the calibration of binder parameters, results of uniaxial compression tests from Table 2 were used for static binder simulations. As cracks were not penetrating the single grains in experiments, only the correct stiffness of aggregates was calibrated, and the bond strength was set to infinity. For the sake of simplicity, the numerical calibration of aggregate parameters was carried out on cylindrical specimens under uniaxial compression. Generally, the model parameters have been calibrated using a gradient-based trial and error principle. The simulations were iteratively repeated using different parameters to minimize the discrepancy between experimental and numerical results. The slope of the stress–strain diagram was used to adjust Young’s modulus of the bonds, while the bond strength parameters were adjusted based on the fracture strength of the macroscopic sample. To estimate an initial parameter set, a linearization-based technique has been used. Here, the strategy based on the direct stiffness method has been applied. The detailed description of this technique is provided in [44].

**Figure 9:**
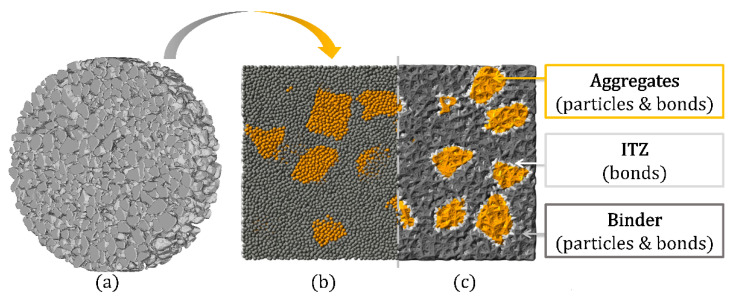
Cross-section detail of the structural three-phase UHPC model: (**a**) Reconstructed 3D models from µCT of aggregate grains; (**b**) Particle view of binder and aggregate without bonds; (**c**) Cross-section showing all particles and bonds.

Finally, using the calibrated parameters for single components such as binder, ITZ, and aggregate, uniaxial static compression of UHPC was simulated and validated with experimental data; see Table 3. Note that the parameters of interparticle friction $\mu_{\mathrm{sl}}$ have not been included in the calibration strategy and were assumed to be equal between all particles. Figure 9 illustrates the cross-section detail of the structural three-phase model, while Table 4 contains the calibrated model parameters.

## 6. Fatigue Simulations

### Uniaxial Fatigue Simulations

**Figure 10:**
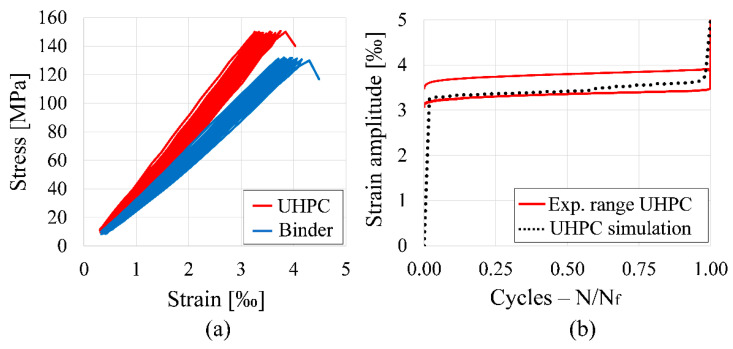
Macroscopic fatigue simulation results of UHPC using the proposed bond formulation with $\delta_{n},\;\delta_{s}=6$: (**a**) Hysteresis loops obtained from fatigue simulations for UHPC and binder; (**b**) Comparison of mean S-N curve from UHPC simulations with experimental range.

After the calibration of model parameters for single components such as binder, ITZ, and aggregate, uniaxial cyclic compression tests were simulated. Three separately generated samples of UHPC and binder were calculated using the same parameter set and validated with experimental results, respectively. The upper and lower load levels ($S_{u}$, $S_{l}$) corresponded to 80% and 5% of the static strength of each simulated cylinder. Figure 10a contains two exemplary hysteresis loops obtained from cyclic UHPC and binder simulations, while Figure 10b shows a comparison between the experimental range of concrete fatigue tests and the mean strain-lifetime (S-N) curve of fatigue UHPC simulations.

By comparing Figure 1a and Figure 10a, it can be clearly noticed that our fatigue simulations are in good agreement with the experimental stress–strain hysteresis loops. The simulations show the same macroscopic characteristics, e.g., the increase of strain and decrease of stiffness until fatigue breakage. Additionally, the mean S-N curve obtained from simulations shows a typical S-curve within the range of the experimental data.

Generally, fatigue simulations were carried out for different damaging factors ($\delta_{n},\;\delta_{s}\;$= 3, 6, 8; Table 5). As already mentioned in Section 5.3, the larger the value for $\delta_{n}$ and $\delta_{s}$, the smaller the represented micro or nanocrack induced in the bond. However, all simulations showed a higher resistance of binder compared with UHPC, which has been equally shown in fatigue experiments. Furthermore, the different damaging factors can be linked to experimental SEM investigations, where a higher content of unhydrated cement clinker leads to higher fatigue resistance (see Section 3.3). The increased presence of unhydrated cement leads to a local increase of packing density; consequently, transverse strain is blocked through the cement particles, and the induced damage is smaller.

**Figure 11:**
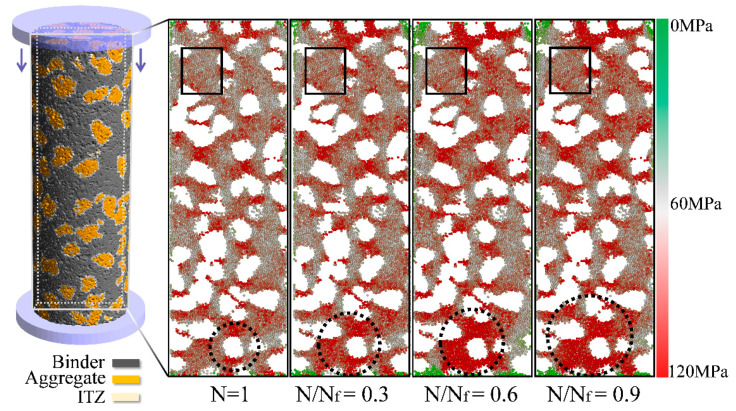
Visualization of stresses on primary binder particles at the upper load level $S_{u}$ and different fatigue stages; stresses are analyzed in the loading direction.

In order to localize the main crack initiation in the investigated UHPC simulations, average stresses on binder particles (at the upper load level) were analyzed at different points of fatigue (N = 1; N/N_f_ = 0.3, 0.6, 0.9). In the first cycle, particles subjected to high stresses were localized between close-knit grains, while the stress exerted on particles is much lower in regions of binder containing no aggregate (Figure 11; N = 1). Aggregate grains absorb inner stresses and contribute to an increase of macroscopic strength. However, discontinuities caused by the high difference of stiffness induce stress peaks and lead to a higher local degradation of the material.

Furthermore, the stress peaks around the aggregate grains propagate into the surrounding matrix with increasing lifetime (dotted circles), while the less stressed regions without grains do not show any significant changes (squares; Figure 11).

**Figure 12:**
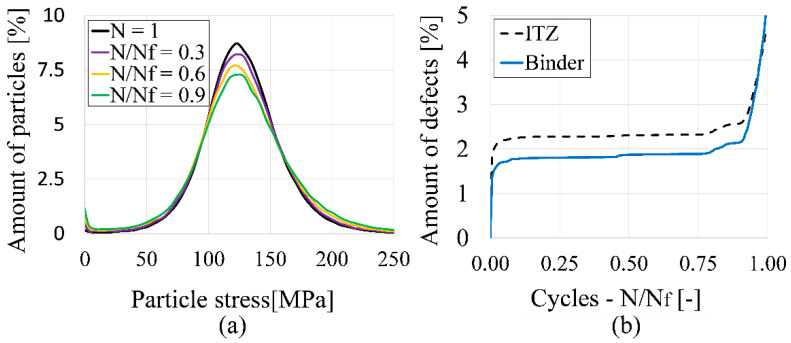
Evaluation of particle stresses and bond failure of the mesoscale model: (**a**) Particle stress distributions of binder particles at different fatigue stages; (**b**) Comparison of ITZ and binder bond failure.

Additionally, average stress distributions on binder particles (in the loading direction) were analyzed at the same fatigue points and load levels (Figure 12a). For higher fatigue stages, the distributions broaden and their maxima drop. This indicates a more inhomogeneous distribution of stress among particles, resulting in the propagation of stress peaks into the surrounding matrix.

Furthermore, the aforementioned stress peaks result in higher stresses within the ITZ bonds and lead to a higher amount of defects during fatigue (Figure 12b). This amount of defects is the ratio of broken bonds compared to its initial number. It can be clearly noticed that the number of broken bonds is dominated by the failure within the ITZ. The largest increase of defects occurs in the first cycles. Afterwards, both curves follow an almost parallel course until breakage. Therefore, the first cycles of fatigue loading play an essential role, as damage and rearrangement within the material structure already occur. Additionally, the binder with its high packing density does not have any buffering effect to reduce stress peaks within the structure, so the damage propagates from the ITZ into the matrix.

## 7. Conclusions

This paper presents an experimental and numerical analysis of the fatigue behavior of UHPC using both scanning and transmission electron microscopy as well as a newly derived bond formulation. Structural inhomogeneities such as local variations in stiffness or ettringite transformation are possible causes for crack initiation. Similar to these inhomogeneities, deterioration can occur on the meso, micro, and nanoscale.

Based on our results, the following main conclusions may be drawn:

- Microscopic investigations proved that fatigue damaging also occurs on the nanoscale as a transformation of nanoscale ettringite. Therefore, to reproduce the correct mechanisms of degradation during cyclic simulations, nanoscale changes must be taken into account even in the case of mesoscale modeling. Using sulfate-resistant cements (SR) containing little or no calcium aluminate could possibly prevent a delayed formation of ettringite and slow down the fatigue process.
- A higher relative content of unhydrated cement clinker in the binder matrix results in higher local strength and consequently leads to a longer specimen lifetime during cyclic loading.
- Stress peaks caused by a high difference of local stiffness manifest in a higher degradation of the binder directly surrounding aggregate grains during fatigue loading. This promotes a crack initiation in the ITZ and favors a propagation in the surrounding matrix. Using less stiff and even more fine-grained aggregate could minimize local stress peaks and increase fatigue lifetime.

For future investigations, UHPC containing fibers or coarse-grained aggregate could be used to bridge the gap between mesoscale and macroscale in both our experimental and numerical studies. 

## Figures and Tables

**Table 1:** Composition for UHPC and binder and physical properties of individual components.

Component	UHPC	Binder	Content [kg/m^3^]	Vol %	Specific Gravity [g/cm^3^]	D50 [μm]
CEM I (52,5)	+	+	795.40	26.16	2.75–3.20	15.49
Silica fume	+	+	168.60	7.80	2.20	*
Quartz powder	+	+	198.40	7.65	2.65	25.07
Quartz sand (0.125/0.5)	+	−	971.00	36.58	2.65	266.98
Superplasticizer (PCE)	+	+	27.80	2.70	1.09	-
Water	+	+	187.90	19.11	1.00	-

* No D50 is available for the used silica fume. Primary grain size is given with 0.1–0.3 μm.

**Table 2:** Mechanical properties of UHPC and binder obtained from experimental tests.

Material	Strength [MPa]	Young’s Modulus [GPa]	Breakage Strain [‰]
UHPC	193.34 ± 3.77	46.67 ± 0.94	4.73 ± 0.26
Binder	172.00 ± 1.63	36.00 ± 1.63	5.33 ± 0.29

**Table 3:** Simulation results obtained from uniaxial compression tests.

Material	Strength [MPa]	Young’s Modulus [GPa]	Breakage Strain [‰]
UHPC	198.12 ± 7.99	45.49 ± 1.14	4.95 ± 1.31
Binder	169 ± 1.67	36.87 ± 0.32	5.21 ± 0.24

**Table 4:** Main material parameters of the BPM model after calibration with static experiments.

Parameter	Binder	ITZ	Aggregate
Particle and bond diameter [mm]	0.48	0.48	0.48
Number of particles [-]	208,125	-	118,687
Number of solid bonds [-]	2,053,425	554,082	1,031,387
Simulations time step [s]	4.00 × 10^−9^	4.00 × 10^−9^	4.00 × 10^−9^
Particle Young’s modulus [GPa]	87.26	-	30.00
Bond Young’s modulus [GPa]	10.00	10.00	40.00
Normal and shear stiffness of particles $k_{n}$, $k_{s}$ [N/m]	8.47 × 10^6^	-	8.47 × 10^6^
Bond normal strength [GPa]	1.08	1.08	∞
Bond shear strength [GPa]	2.81	2.81	∞
Weakening factor α [-]	0.35	0.35	-
Yield limit in tension $\sigma_{\mathrm{ten},\;\mathrm{yield}}$ [MPa]	84.00	84.00	-
Ratio $\sigma_{\mathrm{ten},\;\mathrm{fatigue}}/\sigma_{\mathrm{ten},\;\mathrm{yield}}$ [-]	0.80	0.80	-
Ratio $\tau_{s,\;\mathrm{fatigue}}/\tau_{\max}$ [-]	0.80	0.80	-
Damaging factors $\delta_{s}$, $\delta_{n}$	3.00	3.00	-
Poisson’s ratio [-]	0.19	0.19	0.135
Interparticle sliding friction $\mu_{\mathrm{sl}}$ [-]	0.45	-	0.45

**Table 5:** Average lifetime of UHPC and binder in fatigue simulations with varied damaging factors $\delta_{n}$, $\delta_{s}$.

Material	Average N $\left(\delta_{n},\;\delta_{s}=3\right)$	Average N $\left(\delta_{n},\;\delta_{s}=6\right)$	Average N $\left(\delta_{n},\;\delta_{s}=8\right)$
Binder	156 ± 29	208 ± 22	>300
UHPC	19 ± 6	56 ± 14	161 ± 12

## Supplementary Materials

The following supplementary data are available online at https://www.mdpi.com/article/10.3390/ma14216337/s1. Table S1: Oxide composition of cement and silica fume.
