# Supplementary material for: Discrete Element Modeling and Electron Microscopy Investigation of Fatigue-Induced Microstructural Changes in Ultra-High-Performance Concrete
Source: Materials (Basel). 2021 Oct 23;14(21):6337. doi: 10.3390/ma14216337 (PMC8585305; doi:10.3390/ma14216337)
Supplement: Supplementary file 1 [file materials-14-06337-s001.zip › materials-1418141-supplementary.pdf]

Table S1. Oxide composition of cement and silica fume.

Oxides in cement	Fraction [M.-%]
SiO_2	21.44
Al_2O_3	3.70
Fe_2O_3	4.45
CaO	64.55
MgO	0.77
SO_3	2.48
Na_2O	0.27
K_2O	0.38
**Oxides in silica fume**	**Fraction [M.-%]**
SiO_2	96.00 ± 1.5
